# The Interplay Between Cervicovaginal Microbial Dysbiosis and Cervicovaginal Immunity

**DOI:** 10.3389/fimmu.2022.857299

**Published:** 2022-03-10

**Authors:** Ya Wang, Xiaoli Wang, Meiling Zhu, Li Ge, Xiaochen Liu, Kaikai Su, Zhengzheng Chen, Weidong Zhao

**Affiliations:** Department of Obstetrics and Gynecology, The First Affiliated Hospital of University of Science and Technology of China, Hefei, China

**Keywords:** vaginal, endometrial, female reproductive tract, immunology, microbiota

## Abstract

The cervicovaginal microbiota plays a key role in the health and reproductive outcomes of women. In reality epidemiological studies have demonstrated that there is an association between the structure of cervicovaginal microbiota and reproductive health, although key mechanistic questions regarding these effects remain unanswered and understanding the interplay between the immune system and the structure of the cervicovaginal microbiota. Here, we review existing literature relating to the potential mechanisms underlying the interaction between vaginal microbes and the immune system; we also describe the composition and function of the microbiome and explain the mechanisms underlying the interactions between these microbial communities and various aspects of the immune system. Finally, we also discuss the diseases that are caused by disorders of the reproductive tract and how the immune system is involved. Finally, based on the data presented in this review, the future perspectives in research directions and therapeutic opportunities are explored.

## Introduction

Microbial communities are hypothesized to play an important role in promoting homeostasis. It is known that certain types of cervicovaginal (CV) communities are associated with multitude of adverse outcomes and some CV communities are associated with lower than expected risk of these outcomes. Again, it is important to recognize the difference between causation and association. Compared to other parts of the body, the vagina appears to have a particularly simple and low-diversity microbial community (1). The microbial community in women at childbearing age can be divided into five different categories, referred to as community-state types (CSTs). Four of these CST species are dominated by *Lactobacillus*, namely *L. crispatus* (CST-I), *L. iners* (CST-III), *L. gasseri* (CST-II) and *L. jensenii* (CST-V). The CST-IV category does not feature many *Lactobacillus* species; rather, this category consists of multiple microbial mixtures of strict and facultative anaerobes, including *Gardnerella*, *Atopobium*, *Mobiluncus*, and *Putelltella* (2). There are many kinds of cervicovaginal microbiota, which symbiotic and antagonize each other, and participate in the formation of a complex micro-ecosystem. There is always a dynamic balance between the microbiota and the host, the microbiota and the microbiota, and the microbiota and the environment. This coordinated dynamic balance plays a decisive role in resisting the invasion of pathogenic microorganisms. However, it is hypothesized that the stability of vaginal micro-ecosystems depends on its function and not simply their composition.

Previously, it was assumed that the vaginal microbiota of healthy women was dominated by *Lactobacillus*. However, it is now recognized that the stability of vaginal micro-ecosystems is based on their true function and not simply their composition (3). Evidence shows that the vaginal bacterial community is maintained in a state of dynamic equilibrium and that the vaginal microbiota is affected by personal hygiene, menstruation, hormone levels, and disease states (4). furthermore, Pawel Gajer et al. found that there were five longitudinal patterns of change in vaginal microbial community composition. Moreover, in some women, the vaginal microbial community composition changed markedly and rapidly over time, whereas in others it was relatively stable (5). Disruption of the vaginal ecosystem contributes to the overgrowth of pathogens, thus leading to complex vaginal infections such as bacterial vaginosis (6), sexually transmitted infections (7), and vulvar vaginal candidiasis (8). Interestingly, vaginal microbes can be also used to predict the success of *in vitro* fertilization (9).

## The Uterine Microbiota

Compared to the vaginal microbiota, the upper reproductive tract remains largely unexplored. Previously, the endometrial cavity in healthy women was considered sterile because of the cervical mucus plug. However, the application of next-generation sequencing technologies has increased our perception of the microbiota of the human mucosal surface. Many recent studies have found that certain changes in the uterine microbiota may be related to diseases, such as pelvic inflammation and endometrial cancer (10), and the failure of embryos to undergo implantation (11). For example, Oleer et al. (12)reported the presence of uterine colonies that mainly consisted of Gardenella, Enterobacter bacteria, and Streptococcus lactose. Using 16S rRNA gene sequencing and *Lactobacillus-specific* (*L. iners* & *L*. *crispatus*) *qPCR*, Andrew et al. reported that *Lactobacillus* was rarely found in the endometrium, while the distribution of bacteria in the endometrium and cervix was dominated by *Gardnerella vaginalis, Enterobacter* and *Streptococcus agalactiae* (13).

## Innate Immunity of the Genital Tract (The Mucosal Immune System)

### An Epithelial Barrier in the Mucosa of the Female Genital Tract

The female reproductive tract includes the fallopian tubes, uterus, cervix and vagina. The mucosa of the female reproductive tract varies between the upper and lower reproductive channels. The upper reproductive tract includes the fallopian tube, uterus, and inner cervix, and is covered by a monolayer of columnar epithelium. The lower reproductive tract includes the cervix and vagina; these are covered by a stratified squamous epithelium that forms a more protective barrier than the columnar epithelium. This is a unique system that can balance mucosal immunity to microorganisms and immune tolerance to the sperm, embryo, and fetus (14).

**Figure 1:**
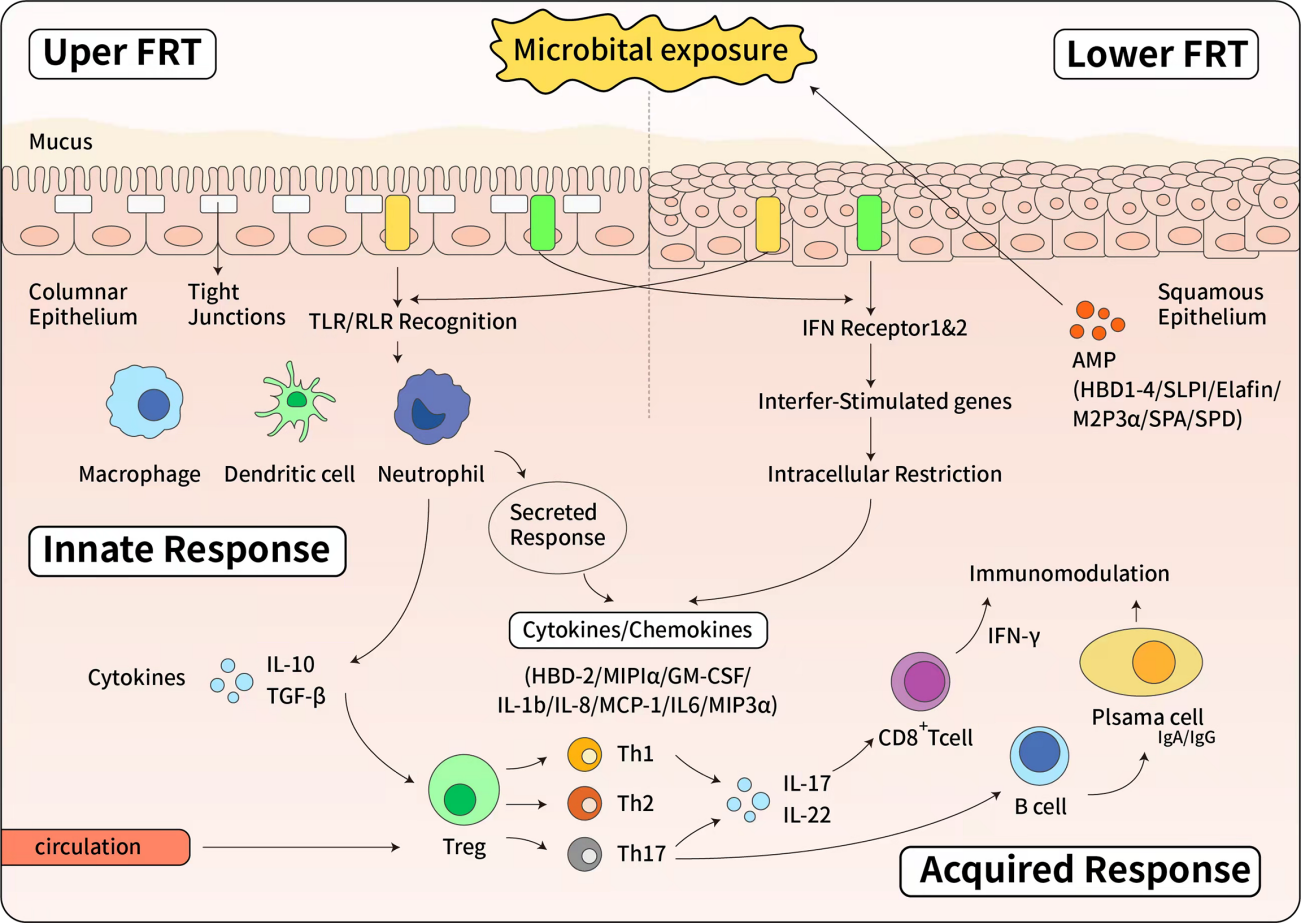
The immune response of female reproductive tract. Upside: The innate immune response of upper female reproductive tract. The upper female reproductive tract, consisting of the Fallopian tubes, uterine endometrium and endocervix is lined by a single layer of columnar epithelial cells linked by tight junctions. The lower female reproductive tract, consisting of the ectocervix and vagina, is covered by a layer of stratified squamous epithelial cells. Below the epithelial layer are innate and adaptive immune cells, as well as some AMPs. When pathogen invades to the epithelial cells, epithelial cells express a panel of Toll-like receptors (TLRs) and RIG-like receptors (RLRs) that can recognize and respond to bacteria or viruses. The Type I interferon (IFN) response is a potent defense system in female reproductive tract cells. Additionally, in response to pathogens, antimicrobials and cytokines/chemokines are secreted to confer broad spectrum protection. Below: The effector of adaptive immune response. Pathogen specific adaptive responses are driven by mucosal macrophages, dendritic cells, and epithelial cells that directly present antigens to T and B cells. Once activated by cytokine stimulation, T and B cells proliferate and differentiate. The cell-mediated response is characterized by the production of IFN and cytotoxic CD8+ T cells that cause apoptosis of infected cells. IFN also stimulates the expression of intracellular antiviral genes that block viral replication. The humoral response is mediated by B cells differentiation into plasma cells that secrete antibodies. Both IgG and IgA are produced in the female reproductive tract and secreted into the mucous.

The mucosal immune system is the first line of defense against viral, bacterial, fungal, and parasitic pathogens (15). In the vagina, the main mucosal cells are the epithelial cells, stromal fibroblasts, and leukocytes; these line the surface of the vaginal mucosa and provide a barrier that controls epithelial cell barrier function (**Figure 1**). Estradiol (E2) increases the proliferation of vaginal epithelium cells, and high levels of progesterone (P4) are associated with vaginal epithelial thinning in animal models, although this has not been observed in humans (16).

Epithelial cells are connected by tight junctions that regulate the movement of molecules through the epithelium. Tight junctions predominate between the basal epithelial cells of the stratified squamous epithelium of the lower female reproductive tract. In contrast, the columnar epithelium in the upper female reproductive tract has a more tightly connected and powerful network (17). A recent study demonstrated that destruction of the epithelium in the female reproductive tract increases the risk of human immunodeficiency virus (HIV) infection by interfering with barrier protection and by promoting the recruitment of HIV target cells. It was shown that the vaginal use of tenofovir (TFV) and tenofovir alafamide (TAF) (a modified TFV prodrug) in HIV prophylaxis trials caused a significant delay in wound closure in the endometrium (EM), endocervix (CX) and ectocervix (ECX). Reconstitution of the tight junctions in epithelial cells of the EM and CX is compromised even after wound closure (18).

A recent study of the mucosal barrier in the reproductive tract showed that the treatment of bovine endometrial epithelial cell lines with astaxanthin (AST, a natural antioxidant carotenoid) reduced the production of lipopolysaccharide-induced interleukin-6 and tumor necrosis factor, increased the activity of cell superoxide dismutase and catalase, and promoted the production of insulin-like growth factor and epithelial growth factor. Furthermore, AST significantly increased the expression of claudin, a tight junction protein that may play an important role in maintaining the host endometrial defense barrier against pathogenic infection. Collectively, these results suggest that AST is a promising agent for endometritis (19).

## Innate Immune Cells

### Uterine Natural Killer (NK) Cells

**Figure 2:**
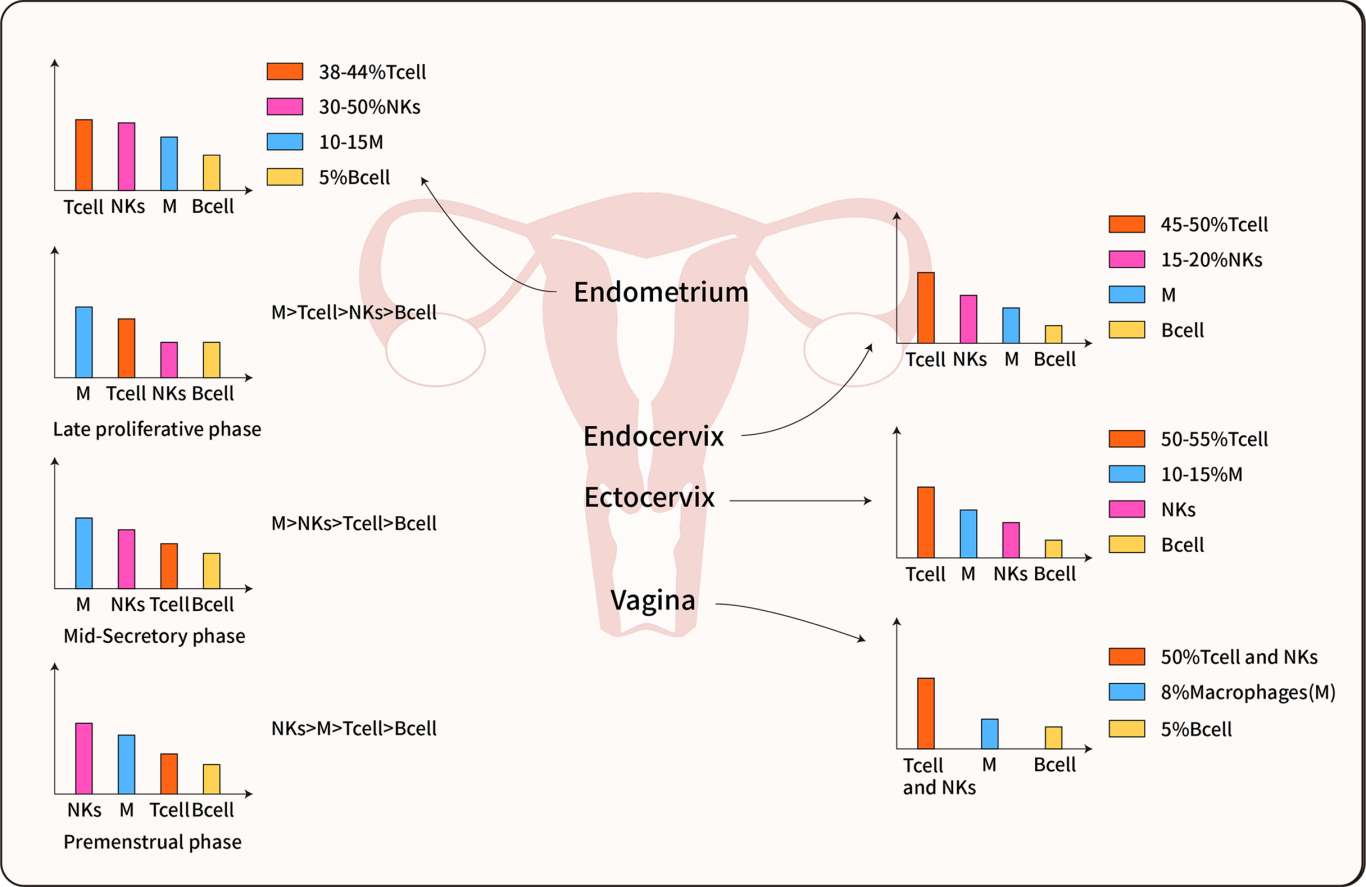
Immune cell distribution in the female reproductive tract. The predominant immune cells are T cells, Macrophages, NK cells, and B cells. The immune cells inconsistent distributed in each organ of the female reproductive tract, furthermore, most data indicate the immune cells also differentially populations in all phases of the menstrual cycle (14, 21, 22).

Human natural killer (NK) cells are a class of innate immune cells that play an important role against pathogenic immunity; this is due to their ability to recognize and lyse infected cells. NK cells are also the dominant form of immune cells at the maternal and fetal interface (20). During the proliferative phase of the endometrium, only a few NK cells are scattered in the matrix of the functional layer. However, during the proliferative phase of the menstrual cycle, only a few NK cells are scattered throughout the stroma of the functional layer. In contrast, there is a dramatic increase in the number of NK after ovulation (**Figure 2**). During the late secretory phase, the number of NK cells surges up (by up to 30-40% of cells) in the stromal compartment and the number of endometrial leukocytes increases up to 70% (17, 23) but numbers of uterine NK cells are thought to reduce in the second half of pregnancy but the mechanism for this reduction is unclear (24).

Three major subpopulations of NK cells have been identified in the decidua based on mRNA expression profiles, cell surface antigens, and metabolic behavior; these subpopulations are referred to as dNK1, dNK2, and dNK3 (25). Recently, Lamond et al. reported that NK cells protect the placenta to avoid invasion by Listeria, an intracellular bacterial pathogen (26). NK cells can also prevent infection-induced abortion *via* the injection of granulysin into the placental trophoblast; this removes intracellular pathogens without damaging placental cells, thus reflecting a mechanism that maintains the tolerance of the maternal-fetal interface to external abnormal factors (20).

### Macrophages

Macrophage are the second most abundant subset of immune cells in the endometrium after uterine NK cells (24). The population of macrophages increases significantly during the secretion phase of the menstrual cycle and accounts for 10–20% of the population of decidual leukocytes (24). Macrophages bind to molecules that are specific to the cell wall of pathogens by specific pattern recognition receptors that participate in the recognition, phagocytosis, and degradation of microbial cells or ‘self’ cells (27). The phage function of macrophages in the female reproductive tract is controlled by dendritic cell-specific regulators that are locally synthesized by cells (e.g., uterine epithelial cells) and regulated by estrogen (28). Recent research suggest that dysregulation of the functions and estrogen responsiveness of female reproductive tract macrophages may be associated with infertility, estrogen- and macrophage-dependent gynecological diseases (29).

### Dendritic Cells

Dendritic cells are a heterogeneous and dynamic population of leukocytes. These are the most potent antigen capture cells (immature dendritic cells) and antigen presentation cells (mature dendritic cells) (30). Recent research demonstrated that uterine dendritic cells exhibit a tolerant phenotype and both uterine dendritic cells and uterine macrophages produce IL-10, TGF-β, and indoleamine 2,3 -dioxygenase, thus helping to maintain a steady state in the microenvironment (31). Dendritic cells in the cervical mucosa can effectively promote the replication of human immunodeficiency virus 1 (HIV-1) and systemic viral dissemination in the cervical mucosa through siglec-1 antibodies (32). A previous study demonstrated that decidualization is a process that involves phenotypic and functional changes of the endometrial stromal cells to sustain immune homeostasis.

## Adaptive Immunity of the Reproductive Tract (Specific Immunity)

### T Cells and B Cells

Adaptive immune cells in the reproductive tract include both B and T lymphocytes. Although B cells are relatively rare in the female reproductive tract, a recent study showed that circulating memory B cells clustered together in a chemokine receptor 3-dependent manner in the vaginal mucosa after secondary infection with herpes simplex virus and then secreted virus-specific IgG2b, IgG2c and IgA into the lumen. This data indicated that circulating memory B cells act as a rapid induction source of mucosal antibodies in the female genital tract (33).

Tissue-resident memory T cells (TRM cells) are composed of both CD4 and CD8 T cell subsets. We found that the distribution of TRM subsets was uneven in the female genital tract with significantly higher levels of CD69^+^CD103^+^CD4 TRM in the vaginal tissue than in cervical tissue (34). Compared to B cells, T cells are always present in the vagina and uterus. Tissue-resident memory T cells in the mucosa of the reproductive tract respond rapidly to reproductive pathogens *via* the innate and adaptive immune systems. In cervical tissues, CD103^+^CD8TRM cells are preferentially localized to the cervical epithelial cells, whereas CD69^+^CD8TRM cells are evenly distributed in the epithelial cells and stroma (35). The production of TRM cells in the vaginal mucosa can provide advanced levels of defense against pathogens. Therefore, T cell-induced vaccines can persistently prevent infections in the mucosa of the reproductive tract, such as HIV (36). Hormone levels are also involved in the regulation of tissue T cells in the female reproductive tract. For example, estradiol treatment for herpes simplex virus 2 in mice led to increased levels of Th1 and Th17 TRM cells in the vagina (37). Estradiol has also been shown to prevent Herpes simplex virus type 2 (38), although the underlying mechanisms remain unknown. Recent studies show that compared with pre-menopausal women, the intra-and extra-cervical CD8^+^ T cells can increase cytotoxic activity in post-menopausal women (39).

## The Secretion of Antimicrobial Peptides (AMP) by the Mucosa of the Reproductive Tract

**Figure 3:**
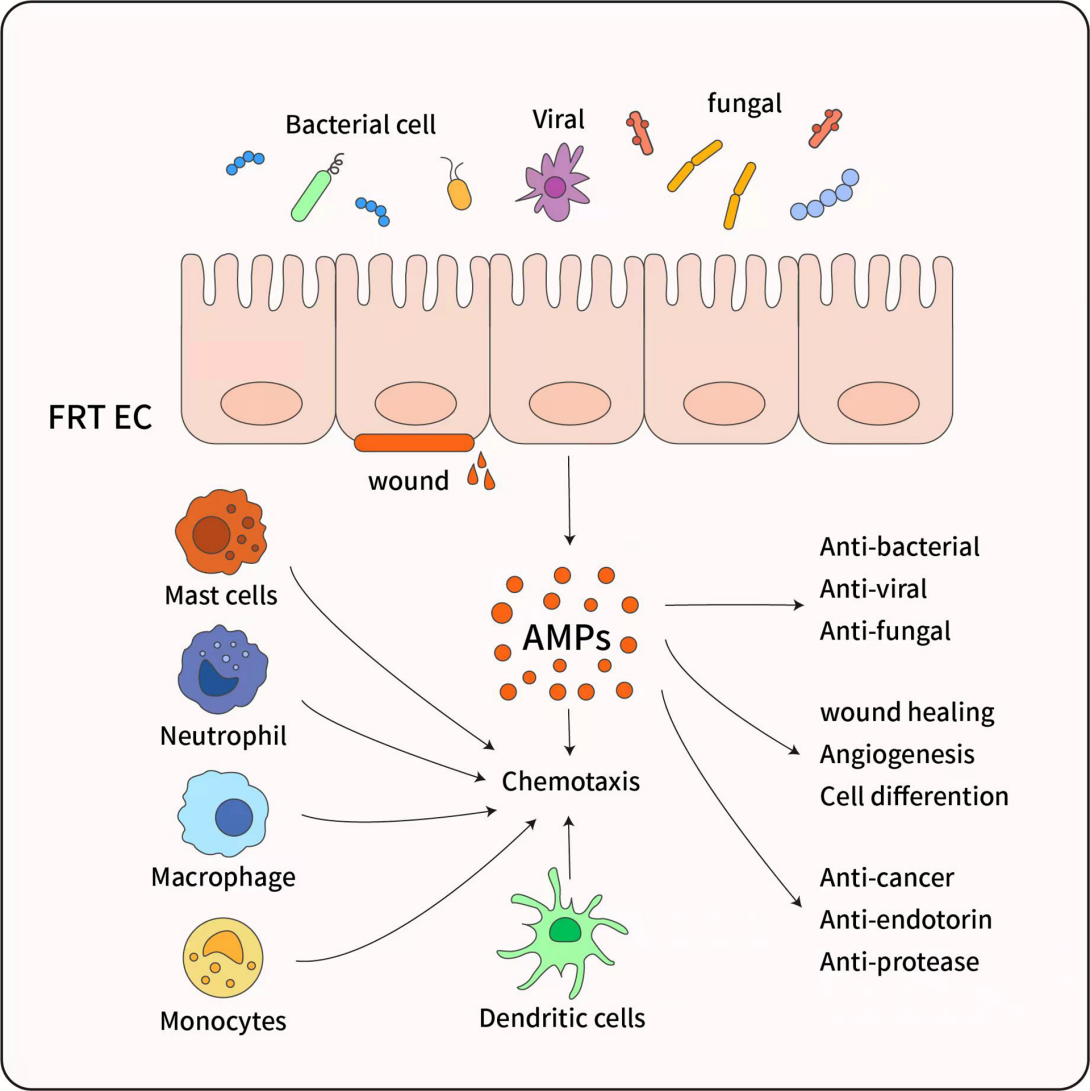
The pleiotropic functions of antimicrobial peptides. AMP have diverse biological effects, which are mainly secreted by female reproductive tract epithelial cells (FRT EC) following exposure to inflammatory or microbial stimuli. AMP have a broad spectrum of activity against bacteria and exhibit anti-fungal and antiviral activity, And AMP promote wound healing and angiogenesis through triggering cell differentiation, ultimate tissue homeostasis is maintained. Although AMP are most recognized for their microbicidal and anti-inflammatory function, AMP also possess immunomodulatory properties through activation of Mast cells, Monocytes/Macrophages, Neutrophils, and Dendritic cells, and inducing chemotaxis to infection sites.

The mucosal surface of the female reproductive tract represents the frontline with regards to defense against microbial challenges from the external environment. Antimicrobial peptides are a class of peptides with both antimicrobial and immunomodulatory properties; these are located at the host barrier. Antimicrobial peptides are effective against bacteria, fungi, enveloped viruses, and protozoa (40) and can even kill tumor cells (**Figure 3**). Importantly, most antimicrobial peptides are non-toxic or less toxic to normal eukaryotic cells and have little pharmacogenetic resistance. Because of this actions, antimicrobial peptides are also known as “endogenous antibiotics”. Antimicrobial peptides are known to protect the reproductive tract and regulate the vaginal microbiome in the lower female reproductive tract to prevent the entry of microbes into the upper female reproductive tract. In addition, antimicrobial peptides can evolve simultaneously with pathogenic lesions (40). Like other tissues, the female reproductive tract has a unique set of antimicrobial peptides that are mainly secreted by epithelial cells (ECs) and neutrophils in the female reproductive tract after exposure to inflammation or microbial stimulation (40). The female reproductive tract expresses a series of AMP, including human beta defensin (HBD), LL -37, SLPI, Elafifin, S100 protein, C-Type lectins, Lysozyme, Iron metabolism proteins, and Kinocidins (**Table 1**). Recent findings indicate the presence of additional antimicrobial peptides in the female reproductive tract (including histone, thrombospondin, lipophilic protein, cystatin A, and ubiquitin) (88), although the complete antimicrobial profile in secretions from the female reproductive tract has yet to be elucidated.

**Table 1:** The identified antimicrobial peptides (AMP) from the mucosa of female reproductive tract.

Endometrium	Reference	Cervix	Reference	Vagina	Reference
CCL20/MIP-3α mRNA	(41)	BPI mRNA	(27)	Calprotectin protein	(42)
CCL20/MIP-3α protein	(28)	BPl protein	(43)	CCL20/MIP-3α mRNA	(44)
Elafin mRNA	(45)	CCL20/MIP-3a mRNA	(44, 46)	CCL20/MIP3α protein	(47)
Elafin protein	(48)	CCL20/MIP-3a protein	(49)	Elafin mRNA	(50)
HBD1 mRNA	(51, 52)	Elafin mRNA	(48)	Elafin protein	(53)
HBD2 mRNA	(54, 55)	Elafin protein	(48)	HBD1 mRNA	(56, 57)
HBD2 protein	(58)	HE4 protein	(59)	HBD1 protein	(57)
HBD3 mRNA	(60)	HBD1 mRNA	(61, 62)	HBD2 mRNA	(63, 64)
HBD4 mRNA	(65, 66)	HBD 2 mRNA	(67)	HBD2 protein	(53)
HE4 protein	(68)	HBD2 protein	(69)	HBD3 mRNA	(70)
Lactoferrin protein	(71)	HBD3 mRNA	(72)	HD5 mRNA	(73)
SLPI mRNA	(74)	HBD3 protein	(75)	HD5 protein	(73)
SLPI protein	(76)	HBD4 mRNA	(77)	HE4 protein	(78)
SP-D mRNA	(79)	HD5 mRNA	(73)	LL -37 mRNA	(77)
SP-D protein	(80)	HD5 protein	(73)	Psorasin protein	(81)
		HD6 mRNA	(73)	SLPI mRNA	(82)
		HNP1-3 protein	(83)	SP -A protein	(84)
		psoriasin protein	(72)	SP -D protein	(85)
		SLPI mRNA	(86)		
		SLPI protein	(86)		
		S P-D RINA	(87)		
		S P-D protein	(87)		

## Cytokines

Inflammatory cytokines can also exert effect on microbes, as confirmed by the presence of specific receptors (89). Cytokines play a unique role in microbial inflammation and can inhibit the growth of Lactobacillus and increase the resistance of this genus of microbes to adverse factors (90). Epithelial cells and potential antigen-presenting cells exert inflammatory responses to *Prevotella, Mobiluncus*, and *Sneathia via* the production of proinflammatory cytokines. For example, IL-1α, IL-1β, and tumor necrosis factor-α (91), along with bacterial vaginosis, may be associated with genetic polymorphisms in the innate immune Toll-like-receptor (TLR1, TLR 2, TLR 4 and TLR 9) and proinflammatory cytokines (IL-1β, IL-1ra, IL-6, IL-6, CXCL8, and IL-10) (92). IL-1 stimulates the resistance of *Lactobacillus* vaginalis to adverse factors, whereas IL-8 and tumor necrosis factor-α primarily increase resistance to peptidoglycans (89).

## Protective Effects of Microbiota in the Reproductive Tract on Hosts

Symbiotic microorganisms are known to interact with the human immune system (93). *Lactobacillus* maintains homeostasis in the reproductive tract to prevent the invasion of pathogens (94). *Lactobacillus* exerts functionality through several mechanisms: (i) by competing for nutrients; (ii) by degrading the glycogen released from vaginal cells to produce organic acids (especially lactic acid) which lowers the vaginal pH, thereby exerting selective antibacterial activity on abnormal microbes; (iii) by producing antimicrobial substances such as bacteriocins and hydrogen peroxide (H_2_O_2_); and (iv) by helping to regulate the local immune system (95). It is important to note that not all *Lactobacillus* species have the same protective capacity; women with a predominant population of inert *Lactobacillus* are known to exhibit higher levels of viral infection (95).

Lactoferrin is a cationic multifunctional glycoprotein that binds iron and plays an important role in immune regulation by exerting antibacterial, antifungal, antiviral, and antiparasitic effects; it can also promote cell growth. When the female genital tract is infected by *Neisseria gonorrhoeae*, *Chlamydia trachomatis*, vaginal trichomas, or vaginal dysregulation, the increased abundance of lactoferrin in the sub-genital mucosa can promote both innate and adaptive immune responses (96). In cases of vaginal dysregulation that are characterized by a small number of vaginal *Lactobacillus* bacteria and an increased number of endogenous anaerobes, the increased abundance of lactoferrin can act as an immunomodulator to maintain the normal healthy microbiota of the vaginal mucosa. Thus, *Lactobacillus* and lactoferrin can be considered as biomarkers of altered microbial homeostasis at the vaginal level. Furthermore, *Lactobacillus* and lactoferrin can be influenced by paracrine activity induced by female hormones and a variety of cytokines. A recent study showed that 17β-estradiol increased adhesion to the vaginal mucosal epithelial cells by altering the morphology of *Lactobacillus crispatus* and inducing the production of biosurfactants (97). Therefore, hormones can be assumed to act as potential mediators to protect or restore vaginal homeostasis.

## Disorders of the Microbiota in the Reproductive Tract Exerts Effect on the Immune System

**Figure 4:**
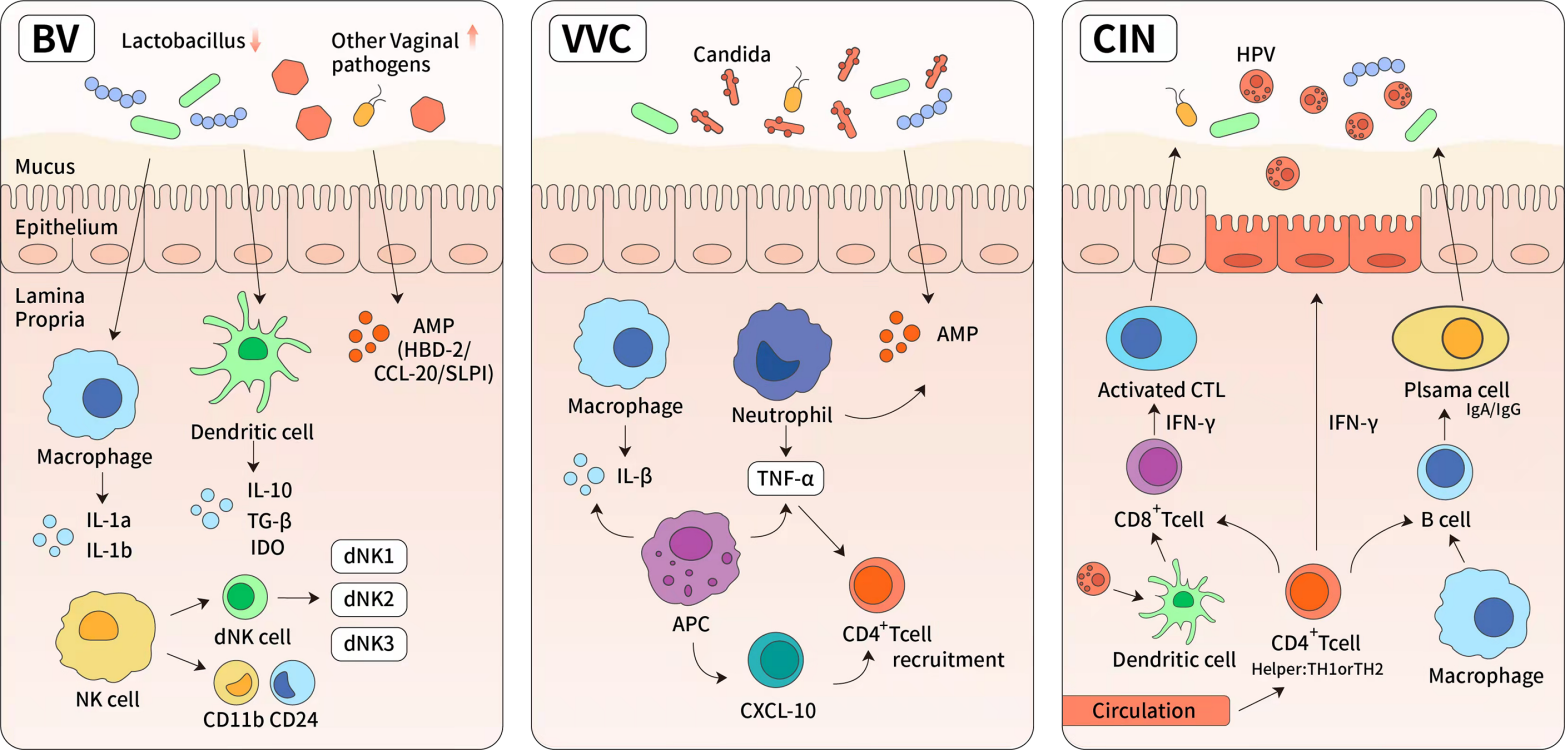
The immune response of reproductive tract diseases. (1) Bacterial vaginosis (BV) is characterized by significant reduction of normal Lactobacillus-dominated microbiota and the overgrowth of anaerobic organisms. These changes lead to higher vaginal pH and increased cytokines, such as IL-8, IL-1a, IL1b, interferon and tumor necrosis factor. (2) Vulvar vaginal candida is predominantly caused by *albicans*. It induces the upregulation of light chain 3, lysosome-associated membrane protein 1, and cytokines (tumor necrosis factor-α and IL-1), then leading to the activation of cellular autophagy. The specific bacterial species were found in highly diverse and Lactobacillus-deficient cervicovaginal bacteria communities. Antigen presentation cells have also been shown to produce chemokine (C-X-C motif) ligand 10 (CXCL10) which can lead to an increase in the number of activated CD4+ T cells. (3) Antivirus-specific immune responses are essential for the eradication of HPV infection, which requires the cooperation of CD4 + Th cells (TH) and cytotoxic CD8+ T cells. It has also been demonstrated that there are numerous CD4+ Th cells and activated TH-1 and TH-2 cells in persistent high-risk human papillomavirus infection. The high levels of interferon-γ secreted by Th-1 cells are known to mediate cytotoxic T cells and directly block viral cytotoxic activity.

The ability of a host to resist pathogenic microorganisms depends on a bidirectional relationship between the immune system and the microbiota (98). Changes in the composition of the vaginal microbiota, even in a small number of microbiota, can induce local immune responses (**Figure 4**). Bacteria related to vaginal dysregulation often produce mucin degradation enzymes (7, 99), induce a pro-inflammatory response (99), damage the mucosal barrier, and promote invasion by sexually transmitted pathogens (7). Disorders of the microbiota in the reproductive tract can also cause inflammatory and non-inflammatory infections in the reproductive tract, especially bacterial vaginitis caused by opportunistic microorganisms, vulvar vaginal candidiasis, and cervical intraepithelial neoplasia (100–102).

### Bacterial Vaginosis

Bacterial vaginosis is characterized by significant reduction of normal *Lactobacillus*-dominated microbiota and the overgrowth of anaerobic organisms (including Gardnerella, Prevotella, and vaginal fungi); collectively, these changes lead to an increase in vaginal pH and foul-smelling secretions. The diversity of microbial communities dominated by bacteria associated with bacterial vaginosis leads to an increase in the levels of cytokines, such as IL-8, IL-1a, IL1b, interferon and tumor necrosis factor (103). In a previous study, antimicrobial peptides of human beta-defensin-2, C-C (cervical cancer) chemokine ligand 20 (CCL20), and secretory leucocyte peptidase inhibitor, were found to be upregulated in cases of bacterial vaginosis, although there was no significant change in CCL20 expression following colonization with *Lactobacillus* species (63). The production of CCL20 is known to be regulated by tumor necrosis factor-α and IL-1β (104). Notably, both CCL20 and HBD-2 encode antimicrobial peptides and ligands for C-C chemokine receptor 6 (CCR6); this is a receptor that is specifically expressed on CD4^+^ T cells, leukocytes, and dendritic cell populations, and regulates the migration of these cells during inflammation. A recent study, based on a three-dimensional human cervical epithelial cell model, found that the expression levels of IL36G were significantly increased in bacterial vaginosis-positive cervical epithelial cells, thus proving that IL-36G is a key regulator of mucosal inflammation, neutrophil transport, and low immunity in the female reproductive tract (105).

### Vulvar Vaginal Candidiasis

Vulvar vaginal candidiasis is the second most common cause of vaginal inflammation and is predominantly causes by *Candida albicans* as the main pathogen. When *Candida albicans* invades the vaginal mucosa, it activates the host innate immune system; this induces the upregulation of light chain 3, lysosome-associated membrane protein 1, and cytokines (tumor necrosis factor-αand IL-1), thus leading to the activation of cellular autophagy (106). The specific bacterial species that were found in highly diverse and *Lactobacillus*-deficient cervicovaginal bacteria communities, were not only associated with sharply elevated levels of genital pro-inflammatory cytokines but also associated with increased genital antigen presentation cell activation through the lipopolysaccharide sensing pathway. Alternatively, antigen presentation cells have also been shown to produce chemokine (C-X-C motif) ligand 10 (CXCL10) which can lead to an increase in the number of activated CD4^+^ T cells (91). Therefore, a small fraction of the vaginal microbiota regulates the local immune system and inflammatory response, thus affecting the susceptibility of infection.

### Cervical Intraepithelial Neoplasia

Disorders of the vaginal microbiota are risk factors for the development of cervical intraepithelial neoplasia. Human papillomavirus infection plays an important role in the etiology and pathogenesis of cervical cancer lesions. Vaginal *Lactobacillus* maintains a low pH environment and produces bacteriocin, thereby maintaining the barrier function of the cervical epithelium to inhibit human papilloma virus (HPV) from entering the basal cells (42). When pathogenic bacteria colonize the epithelium of the reproductive tract, they produce enzymes and metabolites that may impair the barrier and promote the entry of HPV. Recent studies have shown that the risk of developing cervical intraepithelial neoplasia in patients with an abnormal genital microbiota was twice than that in the healthy population (107). The immune response to acute HPV infection was shown to be initially mediated by mucosal NK cells and the production of epithelial antiviral antimicrobial peptides (108). Antivirus-specific immune responses are essential for the eradication of HPV infection; this requires cooperation between CD4 ^+^ Th cells (TH) and cytotoxic CD8^+^ T cells (109). It has also been demonstrated in persistent high-risk human papillomavirus infection, that there are numerous CD4^+^ Th cells, CD25^+^ regulatory T cells, and activated TH-1 and TH-2 cells (110). The high levels of interferon-γsecreted by Th-1 cells are known to mediate cytotoxic T cells and directly block viral cytotoxic activity (111). A previous clinical study confirmed the strong correlation between the Th-1 pattern and the clearance of high risk human papilloma virus (HR-HPV) (112). In contrast, IL-17 has been shown to inhibit immune response effectors in HPV-related diseases (113). The high-risk human papillomavirus has evolved different mechanisms to evade host adaptive responses, including reduced protein secretion or the manipulation of antigen processing machinery (114). The clearance of infection is not a rare event and is often associated with the specific composition of the vaginal microbiota (7).

Microbiota with a reduced content of *Lactobacillus* may contribute to HPV persistence. For example, the prevalence of bacterial vaginosis in women with persistent HR-HPV was reported to be 11%, while the ratio of bacterial vaginosis in women clearing HR-HPV was only 5% (103). Persistent infection with HR-HPV is the leading cause of cervical cancer worldwide (115). Further studies found that HPV infection and/or subsequent clearance was not associated with inflammation or alterations in the subpopulation of cervical T cells but was associated with an increased number of Langerhans cells (116).

## Conclusion

The most important function of the microbiota in the reproductive tract is to maintain immune homeostasis to prevent infection by harmful pathogens. Clinically, external auxiliary factors are used to treat gynecological diseases caused by disorders of the bacterial microbiota, including vaginal acidity, probiotics, hormone therapy and antibiotics. Antibacterial peptides, located on the frontline of the host barrier defense and widely considered as “endogenous antibiotics”, not only prevent host infection by pathogens but can also evolve with pathogens. Most antimicrobial peptides are not toxic or only minimally toxic to normal eukaryotic cells. Therefore, the activation of antibacterial peptides is a strategy to inhibit the pathogenic bacteria to maintain homeostasis in the reproductive tract. Finally, the regulation of cervicovaginal microbiota dysbiosis and immunity may also have important clinical significance and provide new challenges for treating gynecological disease.

## Author Contributions

WZ and ZC planned the project and supervised the overall work. YW and XW wrote the manuscript. LG, MZ, XL, and KS was involved in the article modification. All authors approved the final version to be published.

## Funding

This work was supported by the National Natural Science Foundation of China (82172774), the Fundamental Research Funds for the Central Universities (WK9110000150), and the University Synergy Innovation Program of Anhui Province (GXXT-2019-044).

## Conflict of Interest

The authors declare that the research was conducted in the absence of any commercial or financial relationships that could be construed as a potential conflict of interest.

## Publisher’s Note

All claims expressed in this article are solely those of the authors and do not necessarily represent those of their affiliated organizations, or those of the publisher, the editors and the reviewers. Any product that may be evaluated in this article, or claim that may be made by its manufacturer, is not guaranteed or endorsed by the publisher.
